# Effect of Cavity Cleanser With Long-Term Antibacterial and Anti-Proteolytic Activities on Resin–Dentin Bond Stability

**DOI:** 10.3389/fcimb.2021.784153

**Published:** 2021-11-19

**Authors:** Yaping Gou, Wei Jin, Yanning He, Yu Luo, Ruirui Si, Yuan He, Zhongchi Wang, Jing Li, Bin Liu

**Affiliations:** Key Laboratory of Dental Maxillofacial Reconstruction and Biological Intelligence Manufacturing, Gansu Province, School of Stomatology, Lanzhou University, Lanzhou, China

**Keywords:** antibacterial, endogenous dentin proteases, cavity cleanser, resin–dentin bonds, poly(amidoamine) dendrimers

## Abstract

**Objective:**

Secondary caries caused by oral microbiome dysbiosis and hybrid layer degradation are two important contributors to the poor resin–dentin bond durability. Cavity cleansers with long-term antimicrobial and anti-proteolytic activities are in demand for eliminating bacteria-induced secondary caries and preventing hybrid layers from degradation. The objectives of the present study were to examine the long-term antimicrobial effect and anti-proteolytic potential of poly(amidoamine) dendrimers with amino terminal groups (PAMAM-NH_2_) cavity cleanser.

**Methods:**

Adsorption tests by attenuated total reflectance–infrared (ATR-IR) spectroscopy and confocal laser scanning microscopy (CLSM) were first performed to evaluate whether the PAMAM-NH_2_ cavity cleanser had binding capacity to dentin surface to fulfill its relatively long-term antimicrobial and anti-proteolytic effects. For antibacterial testing, *Streptococcus mutans*, *Actinomyces naeslundii*, and *Enterococcus faecalis* were grown on dentin surfaces, prior to the application of cavity cleanser. Colony-forming unit (CFU) counts and live/dead bacterial staining were performed to assess antibacterial effects. Gelatinolytic activity within the hybrid layers was directly detected by *in situ* zymography. Adhesive permeability of bonded interface and microtensile bond strength were employed to assess whether the PAMAM-NH_2_ cavity cleanser adversely affected resin–dentin bonding. Finally, the cytotoxicity of PAMAM-NH_2_ was evaluated by the Cell Counting Kit-8 (CCK-8) assay.

**Results:**

Adsorption tests demonstrated that the binding capacity of PAMAM-NH_2_ on dentin surface was much stronger than that of 2% chlorhexidine (CHX) because its binding was strong enough to resist phosphate-buffered saline (PBS) washing. Antibacterial testing indicated that PAMAM-NH_2_ significantly inhibited bacteria grown on the dentin discs as compared with the control group (p < 0.05), which was comparable with the antibacterial activity of 2% CHX (p > 0.05). Hybrid layers conditioned with PAMAM-NH_2_ showed significant decrease in gelatin activity as compared with the control group. Furthermore, PAMAM-NH_2_ pretreatment did not adversely affect resin–dentin bonding because it did not decrease adhesive permeability and microtensile strength. CCK-8 assay showed that PAMAM-NH_2_ had low cytotoxicity on human dental pulp cells (HDPCs) and L929.

**Conclusions:**

PAMAM-NH_2_ cavity cleanser developed in this study could provide simultaneous long-term antimicrobial and anti-proteolytic activities for eliminating secondary caries that result from a dysbiosis in the oral microbiome and for preventing hybrid layers from degradation due to its good binding capacity to dentin collagen matrix, which are crucial for the maintenance of resin–dentin bond durability.

## 1 Introduction

Secondary caries caused by oral microbiome dysbiosis and degradation of hybrid layer *via* endogenous dentin proteases are two major challenges encountered in durable resin–dentin bond stability (Gou et al., 2018a). In contemporary minimally invasive dentistry, partial retention of caries-infected dentin is currently recommended to preserve tooth structure and avoid damage to the dental pulp complex (Thompson et al., 2008; Walsh and Brostek, 2013). Nevertheless, entrapped bacteria and their by-products through interfacial gaps between the tooth and the restoration leads to secondary caries that is described as a microbial disease that results from “a dysbiosis in the oral microbiome” (Tanner et al., 2016) and to restoration failure over time (Nyvad and Kilian, 1987; Türkün et al., 2006).

Hybrid layer degradation, caused by hydrolysis of adhesive resin (Breschi et al., 2008; Tjäderhane et al., 2013) and degradation of demineralized collagen matrices in aqueous environments (Mazzoni et al., 2015), is the other challenge in achieving durability of bonds made by resins in dentin. During the acid-etching phase of dentin bonding, endogenous dentin protease such as matrix metalloproteinases (MMPs) and cysteine cathepsins that are normally embedded within the collagen matrix by apatite crystallites become exposed and activated by acid etchants. Subsequent application of acidic resin monomers present in dentin adhesives further promotes activities of these proteases (Tay et al., 2006; Osorio et al., 2011; Frassetto et al., 2016). The activated, matrix-bound protease can progressively degrade denuded collagen fibrils within the hybrid layers, leading to deterioration of resin–dentin bonds over time (Frassetto et al., 2016).

To prolong the durability of resin–dentin interfacial bonds, the aforementioned two challenges should be concomitantly addressed. The use of cavity cleanser with antibacterial and anti-proteolytic properties is in demand. Chlorhexidine (CHX) possesses broad spectrum antibacterial (Twetman, 2004) and anti-proteolytic activities (Scaffa et al., 2012) and has been commonly used as an effective agent to disinfect dentin cavity (Ersin et al., 2008). Nevertheless, CHX is water-soluble and has weak binding affinity for the demineralized dentin collagen matrix (Slee and Tanzer, 1979; Blackburn et al., 2007). It eventually desorbs from the exposed collagen fibrils and slowly leaches out from the hybrid layers over time, thus compromising its long-term antimicrobial and anti-proteolytic effects (Tezvergil-Mutluay et al., 2011).

Recently, poly(amidoamine) dendrimers with amino terminal groups (PAMAM-NH_2_) have been extensively investigated as promising antimicrobial agents due to a great number of positive charges on the protonated amino groups on their exterior (Castonguay et al., 2012; Gou et al., 2017). With numerous positively charged amino groups, PAMAM-NH_2_ is capable of attaching to and puncturing bacteria (Mintzer et al., 2012) cell walls as well as possessing a strong affinity for the denuded dentin collagen (Liang et al., 2015). Hence, it is speculated that when PAMAM-NH_2_ is applied to acid-etched dentin, it would strongly absorb on exposed collagen fibrils to provide relatively long-lasting antimicrobial effectiveness. However, there has been no report whether PAMAM-NH_2_ has inhibitory effects on endogenous dentin proteases.

Accordingly, the objectives of this study were to develop a new dentin cavity cleanser containing PAMAM-NH_2_, to explore its effect of against bacteria grown on dentin surfaces, and to assess the enzyme activity of the resin–dentin interface using *in situ* zymography and functional enzyme activity assays. It was hypothesized that 1) the PAMAM-NH_2_ cavity cleanser has long-term inhibitory effects on bacteria grown on dentin blocks; 2) the PAMAM-NH_2_ cavity cleanser has inhibitory effects on soluble MMP-9 activities; 3) the PAMAM-NH_2_ cavity cleanser has long-term inhibitory effects on endogenous dentin proteases; and 4) treatment of dentin surface with PAMAM-NH_2_ cavity cleanser does not adversely affect dentin bond strength.

## 2 Materials and Methods

PAMAM-NH_2_ utilized in the present work was purchased from ChenYuan Dendrimer Technology Co., Ltd (Weihai, Shandong, China).

### 2.1 Binding Capacity of PAMAM-NH_2_ to Demineralized Dentin

PAMAM-NH_2_ measuring 4 mg/ml and 2% CHX were separately dropped and spread on etched dentin surface using a disposable micro brush. After 60 s of being gently air-dried at room temperature, each dentin surface was subsequently washed with phosphate-buffered saline (PBS) three times and dried again. Attenuated total reflectance–infrared (ATR-IR) spectroscopy (Nicolet iS10, Thermo Scientific, USA) was performed and recorded before and after conditioning with PAMAM-NH_2_ and 2% CHX, and also after washing with PBS. Each group was performed in sextuplicate. Three independent batches were conducted for the experiment.

Fluorescein isothiocyanate (FITC)-labeled PAMAM-NH_2_ was prepared by mixing equimolar amounts of an FITC solution (in acetone) with an aqueous PAMAM-NH_2_ solution overnight in the dark under stirring (Gou et al., 2017). FITC-labeled PAMAM-NH_2_ (4 mg/ml, 50 μl) or FITC was respectively dropped and spread on etched dentin surface using a disposable micro brush. After 60 s, the dentin surface was washed with PBS three times, and the specimens were dried and observed by confocal laser scanning microscopy (CLSM) (Zeiss LSM700, Germany). Quantification of the green fluorescence intensity was calculated with Image-Pro Plus 6.0 (Media Cybernetics, Inc., Silver Spring, MD, USA) to represent the relative binding capacity of PAMAM-NH_2_ to demineralized dentin. Each group was performed in sextuplicate. Three independent batches were conducted for the experiment.

### 2.2 Antibacterial Activity Testing

#### 2.2.1 Bacterial Culture

*Streptococcus mutans* (ATCC 700610), *Actinomyces naeslundii* (ATCC 12104), and *Enterococcus faecalis* (ATCC 29212) were used to examine the antibacterial activities of experimental cavity cleansers. *E. faecalis* was grown aerobically in Brain Heart Infusion (BHI) broth at 37°C. *S. mutans* and *A. naeslundii* were cultured in an anaerobic atmosphere of 5% CO_2_, 90% N_2_, and 5% H_2_ at 37°C in BHI broth. For biofilm formation, the bacteria were cultured in BHI supplemented with 1% sucrose. The bacteria were incubated for 24 h, collected by centrifugation, and rinsed three times with PBS. The bacteria were re-suspended and further diluted in BHI to a final density of 1.0 × 10^7^ colony-forming unit (CFU)/ml. Bacteria density was measured by a microplate reader (Beckman Coulter, Inc., Indianapolis, IN, USA) at the absorbance of 600 nm.

#### 2.2.2 Minimum Inhibitory Concentration

In order to evaluate the effect of PAMAM-NH_2_ on planktonic bacteria, the minimum inhibitory concentration (MIC) was examined by the broth microdilution method. The PAMAM-NH_2_ solution was added in a twofold dilution series in BHI broth in 96-well microtiter plates (1.0 × 10^7^ CFU/ml). After incubation overnight, bacterial growth was measured by a microplate reader at the absorbance of 600 nm. MIC value was determined as the lowest PAMAM-NH_2_ concentration that inhibited at least 90% of bacterial growth compared with the PAMAM-NH_2_-free control. Each group was performed in sextuplicate. Three independent batches were performed for the experiment.

#### 2.2.3 Minimum Biofilm Eradication Concentration

In a physiological state, bacteria tend to exist in biofilms. Bacteria in biofilms are less susceptible to stressful environmental conditions than in their planktonic state. Therefore, minimum biofilm eradication concentration (MBEC) was assessed to evaluate whether PAMAM-NH_2_ has inhibitory effects on biofilms. MBEC was assessed by microtiter plate assay. The testing was started by growing the biofilm first by incubating the suspension of *S. mutans* and *A. naeslundii* for 24 h and *E. faecalis* for 7 days at 37°C. Then, each microplate well was gently washed three times with PBS to remove unattached bacteria, and PAMAM-NH_2_ with different concentrations was added. After that, the microplates were incubated for 24 h (*S. mutans* and *A. naeslundii*) or 7 days (*E. faecalis*) at 37°C and were rinsed with PBS. The biofilm was added with 1% of crystal violet solution and cultured at room temperature. The well was gently washed with PBS for three times to remove excess crystal violet and incubated with 95% ethanol by shaking for 15 min. The optical density at 595 nm was measured with a microplate reader (Model 3550, Bio-Rad). Each group was performed in sextuplicate. Three independent batches were conducted for the experiment.

#### 2.2.4 Cell Counting Kit-8 Counts

*S. mutans*, *A. naeslundii*, or *E. faecalis* biofilm was grown on the surface of dentin discs. Each biofilm-containing dentin disc was conditioned with PAMAM-NH_2_ and 2% CHX and kept for 60 s. The dentin blocks were gently rinsed and transferred into Petri dishes with 1 ml of PBS. The dishes were ultrasonicated to collect biofilms. The collected biofilms were cultured on BHI agar plates, and their viability was assessed by CFU counting after a serial dilution in PBS. Each group was performed in sextuplicate. Three independent batches were conducted for the experiment.

#### 2.2.5 Live/Dead Bacterial Staining

*S. mutans*, *A. naeslundii*, or *E. faecalis* biofilm grown on the surface of dentin discs was treated with a LIVE/DEAD BacLight Bacterial Viability Kit (Molecular Probes, Invitrogen Corp., Carlsbad, CA, USA). Sterilized dentin blocks were incubated with each bacterium at 37°C for 24 h (*S. mutans* and *A. naeslundii*) or 7 days (*E. faecalis*) and were gently washed three times with PBS to remove unattached bacteria. Each biofilm-containing dentin disc was conditioned with PAMAM-NH_2_ and 2% CHX by adding 100 μl of each cavity cleanser onto the dentin surface and kept for 60 s. Then the dentin blocks were treated with 2.5 μM of SYTO 9 and propidium iodide in the dark according to the instructions. Stained dentin blocks were visualized with a CLSM (LSM 780, Carl Zeiss, Oberkochen, Germany) equipped with a 20× objective lens by the channel set at excitation/emission wavelengths 480/500 nm for SYTO 9, and 590/635 nm for propidium iodide. Live bacteria are dyed green, dead bacteria are dyed red, and adjacent live and dead bacteria are shown as yellow when they are merged. Quantification of dead and live bacteria was calculated based on the value of relative green and red fluorescence by Image-Pro Plus 6.0 software. Each group was performed in sextuplicate. Three independent batches were conducted for the experiment.

### 2.3 Analysis of the Effect of PAMAM-NH_2_ on Matrix Metalloproteinases

#### 2.3.1 Inhibition of Soluble rhMMP-9

The inhibitory effects of PAMAM-NH_2_ and 2% CHX on soluble purified recombinant human (rh) MMP-9 were assessed using purified rhMMP-9 (AS-55576) and the Sensolyte Generic MMP assay kit (AS-72095) (all from AnaSpec Inc., CA, USA). The MMP assay kit contains an intact substrate (thiopeptolide) that is disintegrated by MMPs to release a colored product, 2-nitro-5-thiobenzoic acid.

A series of PAMAM-NH_2_ solutions (0.5, 1, 2, 4, 8, and 16 mg/ml) were prepared as test agents. The substrate solution provided by the assay kit was prepared at 0.2 mM. In the experimental groups, the well contained 2 μl of rhMMP-9, 50 μl of substrate solution, and 10 μl of potential MMP inhibitor.

The control groups included 1) the positive control group: rhMMP-9 enzyme only without the anti-MMP agent; 2) the inhibitor control group: rhMMP-9 enzyme and MMP inhibitor (GM6001); 3) the test compound control group: assay buffer and various concentrations of PAMAM-NH_2_ solutions; and 4) the substrate control group: assay buffer only. The reagents on the plate were shaken for 30 s to mix completely. Readings were measured every 10 min for 60 min. The intensity of color was detected using a microplate reader at 412 nm. The potency of MMP-9 inhibition by GM6001 (known MMP inhibitor) and the six concentrations of PAMAM-NH_2_ were exhibited as percentages of the adjusted absorbance of the “positive control.” Percent inhibition of the MMP (%) was calculated as 1 − ([A]_test compound group_ − [A]_test compound control_)/([A]_positive control_ − [A]_substrate control_), where [A] represents the absorbance values of the wells. Each specimen was performed in sextuplicate (N = 6). Three independent batches were conducted for the experiment.

#### 2.3.2 *In Situ* Zymography

Ten teeth from two cavity cleanser group were used for *in situ* zymography of the bonded interface. After being treated with 37% phosphoric acid, the dentin blocks were conditioned with each cavity cleanser for 60 s and gently air-dried. After being bonded with adhesive, a 2-mm-thick layer of flowable resin composite was placed and light-cured. After 24 h of storage in deionized water, the bonded samples were cut vertically into 1-mm-thick slabs to expose the resin–dentin interface.

The bonded slab was fixed to a glass slide and polished to approximately 50-μm thickness. The EnzChek™ Gelatinase/Collagenase Assay Kit (E-12055, Molecular Probes, Eugene, OR, USA) was employed for *in situ* zymography to identify sites of MMP activity within the hybrid layers. Briefly, the 1.0 mg/ml stock solution of self-quenched fluorescein-conjugated gelatin was diluted by adding deionized water and mixing with anti-fading agent. Then, 50 μl of the self-quenched fluorescent gelatin mixture was dropped on top of each bonded slab and covered with a coverslip. The slides were kept away from light and incubated in a humidity chamber at 37°C for 48 h.

A two-photon CLSM (LSM 780, Carl Zeiss, Thornwood, NY, USA) was used to acquire images using a 40× oil immersion objective lens, with the channels set at 488/530 nm (excitation/emission wavelengths). Green fluorescence derived from de-quenched fluorescein released from disintegrated gelatin was imaged. Sections that were 85 μm thick were acquired from different focal planes of each bonded specimen. The images were stacked and processed with ZEN 2010 software (Carl Zeiss). The image-Pro Plus 6.0 software was employed to quantify the green fluorescence intensity. Each group was performed in sextuplicate. Three independent batches were conducted for the experiment.

### 2.4 Assessment of the Impact of PAMAM-NH_2_ on Resin–Dentin Bonding

#### 2.4.1 Adhesive Permeation of Bonded Interface

Twenty freshly extracted and intact human third molars were collected for permeability evaluation of adhesive. The dentin was sectioned at a distance of 2.5 mm away from the deepest pulpal horn using a slow-speed saw under water cooling. One drop of fluorescein sodium (Sigma-Aldrich, St Louis, MO, USA) was mixed with three drops of adhesive (Prime & Bond^®^ NTTM) to produce a fluorescent adhesive. The bonded dentin was glued to fenestrated Perspex discs with cyanocrylate glue. The assembly was connected *via* an 18-gauge stainless steel tube to fenestrated Perspex discs. The latter was placed to a column of 0.1% green fluorescent dye solution (Alexa Fluor™ 405, excitation/emission: 401/421 nm; Thermo Fisher Scientific) oriented 20 cm above the Plexiglass block to simulate pulpal pressure. This generated water pressure through the dentinal tubules during the acid-etching process, treatment with each cavity cleanser, bonding, and resin composite buildup. The setup was kept away from light and incubated for 4 h to enable water to continue permeating the bonded interface.

After pressure perfusion, the bonded specimen was removed from the fenestrated Perspex discs and cut vertically to get a 1-mm slab containing the water perfused bonded interface. Each bonded specimen was fixed to a glass slide and polished to approximately 50-μm thickness. A two-photon CLSM was used to acquire images using a 40× oil immersion objective lens. Green fluorescence was imaged together with the red fluorescence derived from dyed adhesive. Sections that were 85 μm thick were acquired from different focal planes of each bonded specimen. The images were stacked and processed with ZEN 2010 software (Carl Zeiss). The image-Pro Plus 6.0 software was employed to quantify the dyed adhesive permeation. Each group was performed in sextuplicate. Three independent batches were conducted for the experiment.

#### 2.4.2 Bond Strengths to Dentin

Thirty human third molars were collected for bond strength test. The teeth were cut 2–3 mm below the cementoenamel junction to remove roots using water-cooled low-speed cutting saw (Isomet, Buehler Ltd., Lake Bluff, IL, USA). The occlusal enamel was removed perpendicular to the longitudinal axis of each tooth to expose flat midcoronal dentin surface. The exposed midcoronal dentin surface was polished with 600-grit silicon carbide paper under water for 60 s to produce a standardized smear layer.

Exposed dentin surfaces were randomly allocated to two groups according to the adhesives used: Prime & Bond^®^ NT™ (PB, Dentsply DeTrey, Konstanz, Germany) and Adper™ Single Bond Plus (SBP, 3M ESPE, St. Paul, MN, USA). Each specimen was treated with 37% phosphoric acid (Uni-Etch, Bisco Inc., Schaumburg, IL, USA) for 15 s, washed with deionized water for 15 s, and air-dried for 5 s. Dentin surface from each adhesive group was further randomly assigned to one of the following three subgroups for dentin pretreatment with PAMAM-NH_2_, 2% CHX, and deionized water (control) (N = 6). The etched dentin surface was pretreated with respective cavity cleanser or deionized water for 60 s and air-dried for 5 s. The adhesives were placed to the etched dentin and light-cured for 15 s with a light-curing unit. Resin composite buildups (Z250, 3 M ESPE, St. Paul, MN, USA) were constructed.

After storage in deionized water for 24 h, the bonded teeth were subsequently sectioned vertically to 0.9-mm-thick slabs using a low-speed saw under water cooling. The slabs were further cut into 0.9 mm × 0.9 mm × 8 mm long beams. Each beam was attached and stressed to failure under tension using a universal tensile testing machine (HD-B609B-S, Haida International, China). Each specimen was performed in sextuplicate. Three independent batches were conducted for the experiment.

After tensile bond strength test, the dentin side of the fractured beams was detected using a stereoscopical microscope at ×40 magnification to determine the failure mode. Failure modes were classified as adhesive failure (A), mixed failure (M, failure extending into dentin or resin composite), cohesive failure in resin composite (CC), and cohesive failure in dentin (CD).

### 2.5 Cytotoxicity Assay

Human dental pulp cells (HDPCs) and mouse fibroblast cells L929 were chosen to test the cytotoxicity of PAMAM-NH_2_. Freshly extracted and intact human third molars were collected, cleaned, and cut perpendicular to the longitudinal axis of each tooth to expose the pulp chamber, with the donors’ written informed consent. Dental pulp tissues were gently removed by blunt non-cutting forceps and dispersed in 2 mg/ml of collagenase/dispase for 1.5 h to retrieve HDPCs at 37°C. The HDPCs and L929 were cultured in Dulbecco’s Modified Eagle’s Medium plus 10% heat-inactivated fetal bovine serum and 1% penicillin/streptomycin at 37°C in a humidified incubator supplemented with 5% CO_2_. The seeded cells were subsequently cultured until 80% confluency was achieved. Four passage cells were used for the experiment.

The cytotoxicity of PAMAM-NH_2_ was determined by the Cell Counting Kit-8 (CCK-8) assay. Cells were seeded in a 96-well microtiter plate at a density of 1.0 × 10^4^ cells/well and incubated overnight. The culture medium was replaced with 100 μl of fresh culture medium containing different concentrations of PAMAM-NH_2_. After incubation overnight, 10 μl of CCK-8 solution was added to each well, and the microtiter plate was incubated at 37°C for 4 h, after which the absorbance at 570 nm was determined with a microplate reader (Spectra Plus, Tecan, Zurich, Switzerland). The cell viability (%) = ([A]_test_ − [A]_blank_)/([A]_control_ − [A]_blank_) × 100%, where [A]_test_, [A]_control_, and [A]_blank_ represent the absorbance values of the wells with cells and PAMAM-NH_2_, those with cells and without PAMAM-NH_2_, and those without cells or PAMAM-NH_2_, respectively. For each sample, the average absorbance from six wells that run in parallel was calculated. Three independent batches were conducted for the experiment.

### 2.6 Statistical Analyses

Data were expressed as means and SDs. For each parameter to be analyzed, the data sets were evaluated for their normality (Shapiro–Wilk test) and equal variance assumptions (modified Levene’s test) before use of parametric statistical methods. If those assumptions were not violated, the data sets were analyzed with one-factor ANOVA or one-factor repeated-measures ANOVA, depending on the parameter tested. Post-hoc comparisons were conducted using the Holm–Sidak procedures to identify statistical significance among groups. If the assumptions were violated, the data sets were non-linearly transformed to satisfy those assumptions before performing the aforementioned statistical procedures. For all tests, statistical significance was set at α = 0.05.

## 3 Results

### 3.1 Binding Capacity of PAMAM-NH_2_ to Demineralized Dentin

**Figure 1:**
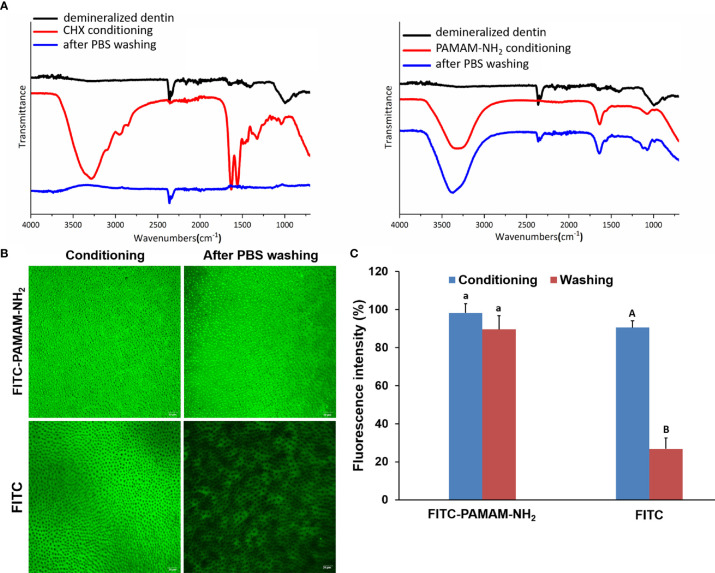
The binding capacity of PAMAM-NH_2_ to demineralized dentin. **(A)** The ATR-IR spectrum of the demineralized dentin, after conditioning with CHX or PAMAM-NH_2_ and after washing with PBS. **(B)** The CLSM images of the demineralized dentin immobilized with FITC-labeled PAMAM-NH_2_ or free FITC, after washing with PBS. **(C)** Bar graph of relative fluorescence intensity of the two groups. Data are means ± SDs. Data obtained in the three groups (N = 6/group) were analyzed. For FITC-PAMAM-NH_2_, columns labeled with the same lowercase letters are not significantly different (p > 0.05). For FITC, columns labeled with different uppercase letters are significantly different (p < 0.05). ATR-IR, attenuated total reflectance–infrared; PBS, phosphate-buffered saline; CLSM, confocal laser scanning microscopy; FITC, fluorescein isothiocyanate.

The ATR–Fourier-transform IR (ATR-FTIR) spectra of the demineralized dentin after being conditioned with 4 mg/ml of PAMAM-NH_2_ or 2% CHX and after being washed with PBS are shown in **Figure 1A**. The peak at 1,000.1 cm^−1^ was due to phosphate v1, v3 functional groups, which is a characteristic peak of dentin. After being conditioned with PAMAM-NH_2_, characteristic peaks of PAMAM-NH_2_ were clearly detected at 3,088.52 to 3,500 cm^−1^ corresponding to amide vibration and at 1,634.9 cm^−1^ corresponding to amide carbonyl groups, and they are related to the number of amide groups in the branches of PAMAM-NH_2_. The results confirmed that PAMAM-NH_2_ bound to the demineralized dentin. After thorough washing with PBS, these characteristic peaks of PAMAM-NH_2_ were still apparent, which indicated that PAMAM-NH_2_ can resist PBS washing. After being conditioned with CHX, the presence of characteristic peaks of CHX was detected, as well as C–H_2_ stretches at 2,948.5 and 2,855.0 cm^−1^, C═N vibration at 3,319.1 cm^−1^, and para-substitution of benzene rings at 1,631.5 and 1,557.0 cm^−1^. Following PBS washing, these characteristic peaks disappeared in CHX conditioning dentin surface, indicating that CHX was washed off by PBS.

FITC-labeled PAMAM-NH_2_ and free FITC were dropped onto demineralized dentin surface. After being air-dried at room temperature, the dentin surfaces were rinsed with PBS and observed by CLSM. **Figure 1B** shows yellow-green fluorescence, which could be clearly observed for both two groups, with an intensity value of 98.2% ± 4.9% for the FITC-PAMAM-NH_2_ group and 90.5% ± 7.1% for the FITC group (**Figure 1C**). However, little fluorescence could be detected on free FITC-coated dentin surface after PBS washing (**Figure 1B**) reaching 26.8% ± 5.7% fluorescence intensity, which was significantly lower than that of the FITC group before PBS washing (**Figure 1C**, p < 0.05). In contrast, FITC-PAMAM-NH_2_-coated dentin retained most of the fluorescence with an intensity value of 89.6% ± 3.5% (**Figures 1B, C**). There was no significant difference in the fluorescence intensity in the FITC-PAMAM-NH_2_ group before and after PBS washing (p > 0.05). This result demonstrated that free FITC could not bind to the demineralized dentin surface and that PAMAM-NH_2_ had a good binding capacity to demineralize dentin.

### 3.2 Antibacterial Activities

**Figure 2:**
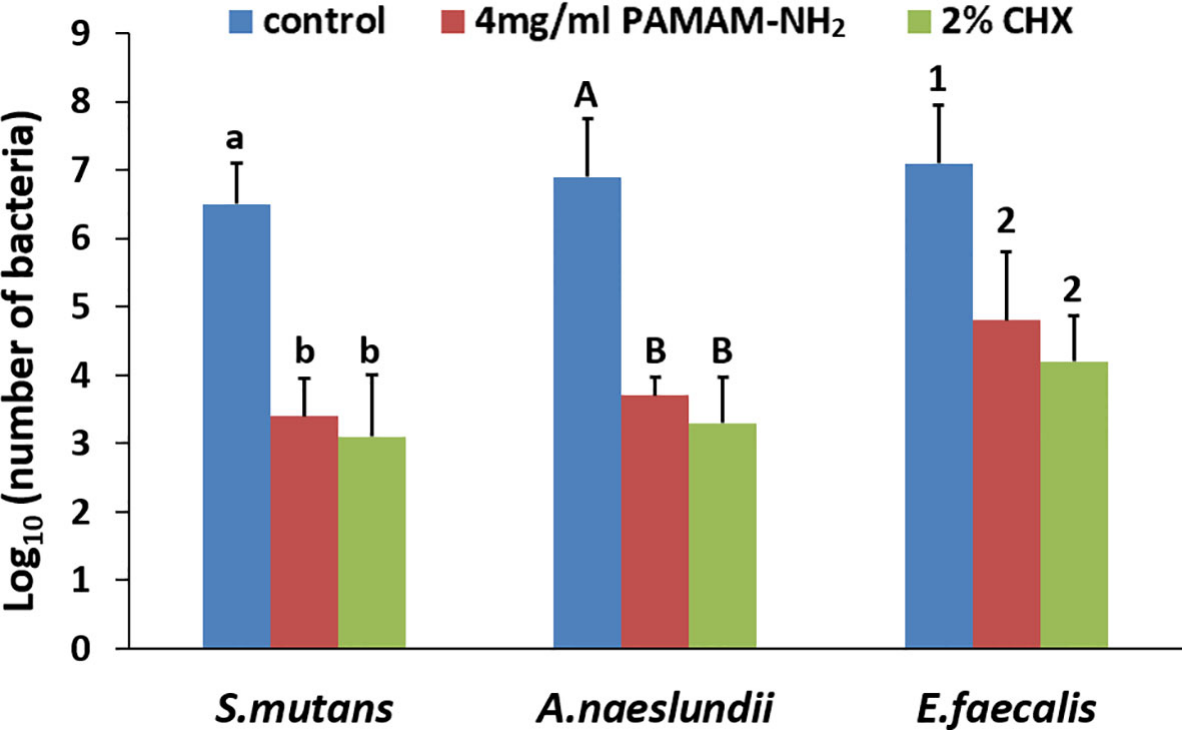
CFU counts of *Streptococcus mutans*, *Actinomyces naeslundii*, or *Enterococcus faecalis* grown on dentin blocks for the deionized water control and the two cavity cleansers groups. Values are mean and SDs. Data obtained in the three groups (N = 6/group) were analyzed. For each bacterium strain, columns labeled with different letters or numbers are significantly different (p < 0.05). CFU, colony-forming unit.

A microdilution method was used to assess the antibacterial activity of PAMAM-NH_2_. MIC values of PAMAM-NH_2_ required to inhibit the visible growth of planktonic bacteria were 562.5, 562.5, and 750 μg/ml for *S. mutans*, *A. naeslundii*, and *E. faecalis*. MBEC values of PAMAM-NH_2_ on these three bacteria were 750, 1,125, and 3,500 μg/ml (**Table 1**). **Figure 2** shows the CFU counts of *S. mutans*, *A. naeslundii*, and *E. faecalis* biofilms after treatment with deionized water, PAMAM-NH_2_, or 2% CHX. For these three bacteria, control dentin blocks (without cavity cleanser) all had the highest CFU. Dentin blocks treated with the PAMAM-NH_2_ and 2% CHX significantly reduced viable bacteria in the biofilms, compared with dentin blocks without cavity cleanser (p < 0.05). No significant difference was found between the PAMAM-NH_2_ and the 2% CHX (p > 0.05). These results showed that PAMAM-NH_2_ cavity cleanser had inhibitory effects on bacteria grown on dentin blocks.

**Table 1:** MIC and MBEC values of PAMAM-NH_2_ against Streptococcus mutans, Actinomyces naeslundii, and Enterococcus faecalis.

Bacteria	MIC (μg/ml)	MBEC (μg/ml)
*S. mutans*	562.5	750
*A. naeslundii*	562.5	1,125
*E. faecalis*	750	3,500

MIC, minimum inhibitory concentration; MBEC, minimum biofilm eradication concentration.

**Figure 3:**
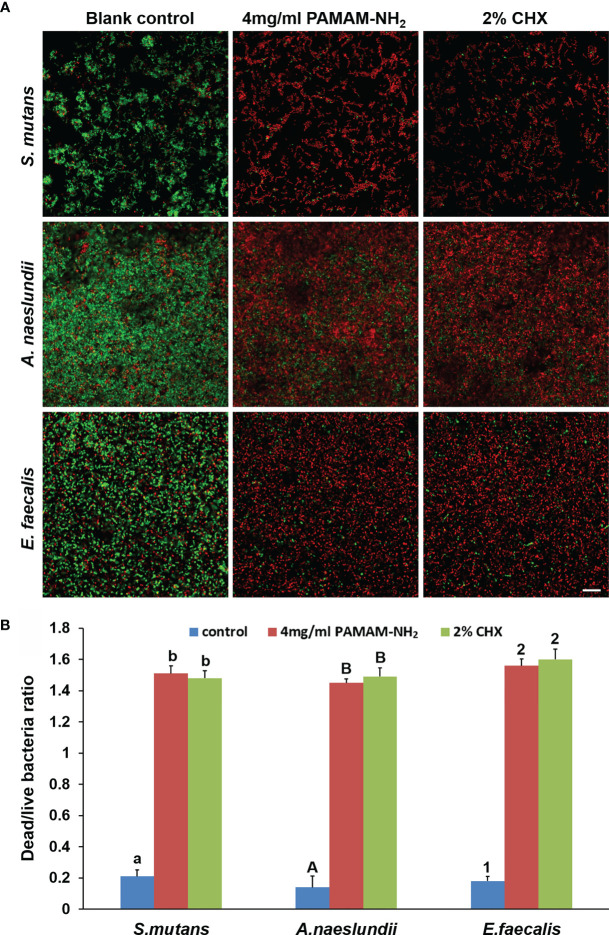
**(A)** Representative CLSM images of *Streptococcus mutans*, *Actinomyces naeslundii*, or *Enterococcus faecalis* (live, green; dead, red) grown on dentin blocks after application of deionized water (control), and 4 mg/ml of PAMAM-NH_2_ or 2% CHX as cavity cleansers. Bars = 20 μm. **(B)** Bar chart of the dead/live bacteria ratio of the three groups based on analysis of the live–dead staining profiles of the dentin blocks. Values are means and SDs. Data obtained in the three groups (N = 6/group) were analyzed. For each bacterium strain, columns labeled with different letters or numbers are significantly different (p < 0.05). CLSM, confocal laser scanning microscopy

**Figure 3A** shows representative CLSM images of the distribution of *S. mutans*, *A. naeslundii*, and *E. faecalis* biofilms stained with live/dead stains. For all *S. mutans*, *A. naeslundii*, and *E. faecalis*, biofilms grown on control dentin blocks (without cavity cleanser) consisted of primarily live bacteria, with small amounts of dead bacteria. In contrast, biofilms in the PAMAM-NH_2_ and 2% CHX dentin blocks showed primarily dead bacteria and a higher dead/live bacteria ratio as compared with the control group (p < 0.05, **Figure 3B**), which indicates that PAMAM-NH_2_- and 2% CHX-pretreated dentin possessed antimicrobial activity. There was no significant difference in the antibacterial effect between the PAMAM-NH_2_ and 2% CHX groups (p > 0.05, **Figure 3B**).

### 3.3 Inhibitory Effect of PAMAM-NH_2_ on Matrix Metalloproteinases

**Figure 4:**
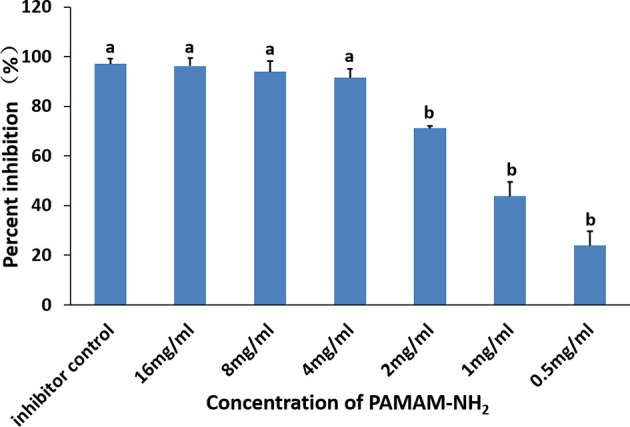
Inhibitory effect of PAMAM-NH_2_ concentration on soluble rhMMP-9. Data are means ± SDs. Data obtained in the three groups (N = 6/group) were analyzed. Columns identified with the different lowercase letters are significantly different (p < 0.05).

The inhibitory effects of different concentrations of PAMAM-NH_2_ on soluble MMPs are shown in **Figure 4**. The relative percentages of rhMMP-9 inhibitor by the GM6001 (kit inhibitor control) were 97.02% ± 2.10%. Inhibition of rhMMP-9 by PAMAM-NH_2_ at concentrations higher than 4 mg/ml (4 to 16 mg/ml) was comparable with that of the GM6001 group (inhibitor control) (p > 0.05). The anti-MMP activities of PAMAM-NH_2_ at concentrations ranging from 0.5 to 2 mg/ml were significantly lower than those of the kit inhibitor control (p < 0.05).

**Figure 5:**
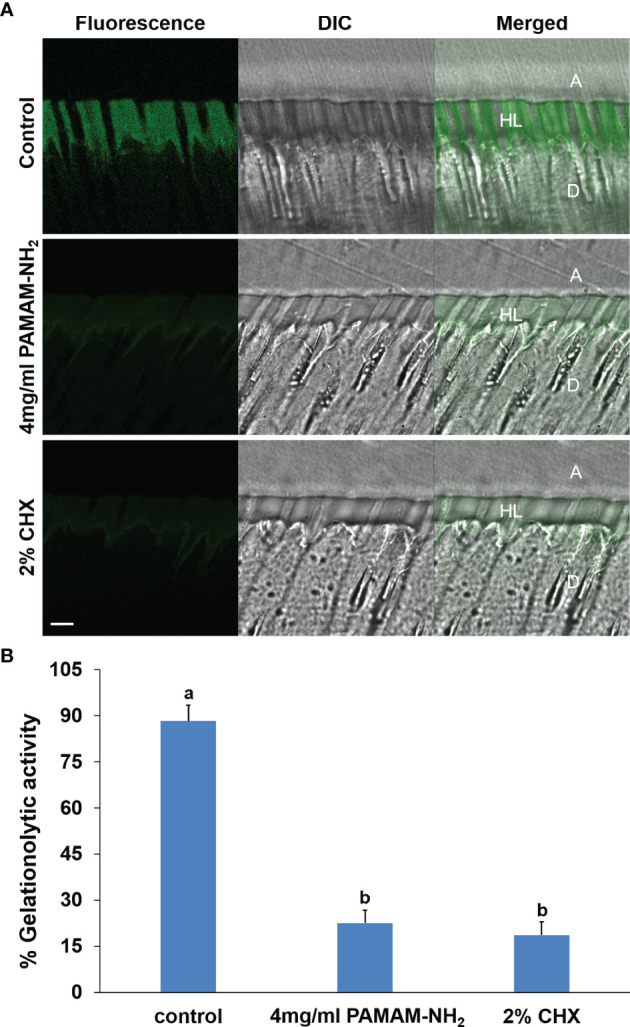
**(A)** Representative CLSM images of *in situ* zymography performed in resin–dentin interfaces pretreated with the deionized water control, 4 mg/ml PAMAM-NH_2_ cavity cleanser, or the 2% CHX cavity cleanser prior to adhesive application. Bars = 5 μm. A, adhesive layer; HL, hybrid layer; D, dentin. Green channel: fluorescence derived from dequenched fluorescein released after breaking down of the highly quenched fluorescein-conjugated extrinsic gelatin source into smaller peptides. DIC, differential interference contrast image of the resin–dentin interface. **(B)** Quantified *in situ* zymography data depicting the percentage of hybrid layers that exhibit activity against extrinsic fluorescein-conjugated gelatin in the deionized water control, 4 mg/ml of PAMAM-NH_2_ cavity cleanser, or the 2% CHX cavity cleanser. Data are means ± SDs. Data obtained in the three groups (N = 6/group) were analyzed. Columns labeled with different lowercase letters are significantly different (p < 0.05). CLSM, confocal laser scanning microscopy.

The *in situ* zymography technique enables screening of the relative proteolytic activities directly within dentin hybrid layers. Representative CLSM images of dentin pretreated with deionized water (control), PAMAM-NH_2_, and 2% CHX are shown in **Figure 5A**. **Figure 5B** summarizes the relative percentage areas of hybrid layers in the three groups that showed green fluorescence after coming in contact with the highly quenched fluorescein-conjugated gelatin. For the control dentin slabs pretreated with deionized water, an intense green fluorescence was detected within the hybrid layers, reaching 88.3% ± 5.2% fluorescence intensity. In contrast, the HLs in the experimental groups pretreated with 4 mg/ml of PAMAM-NH_2_ and 2% CHX showed weak green fluorescence, with an intensity value of 22.6% ± 4.1% and 18.6% ± 4.4%, respectively. These fluorescence values were significantly lower than those of the control group (p < 0.05). No significant difference was found between the PAMAM-NH_2_ group and the 2% CHX group (p > 0.05).

### 3.4 Assessment of the Impact of PAMAM-NH_2_ on Resin–Dentin Bonding

**Figure 6:**
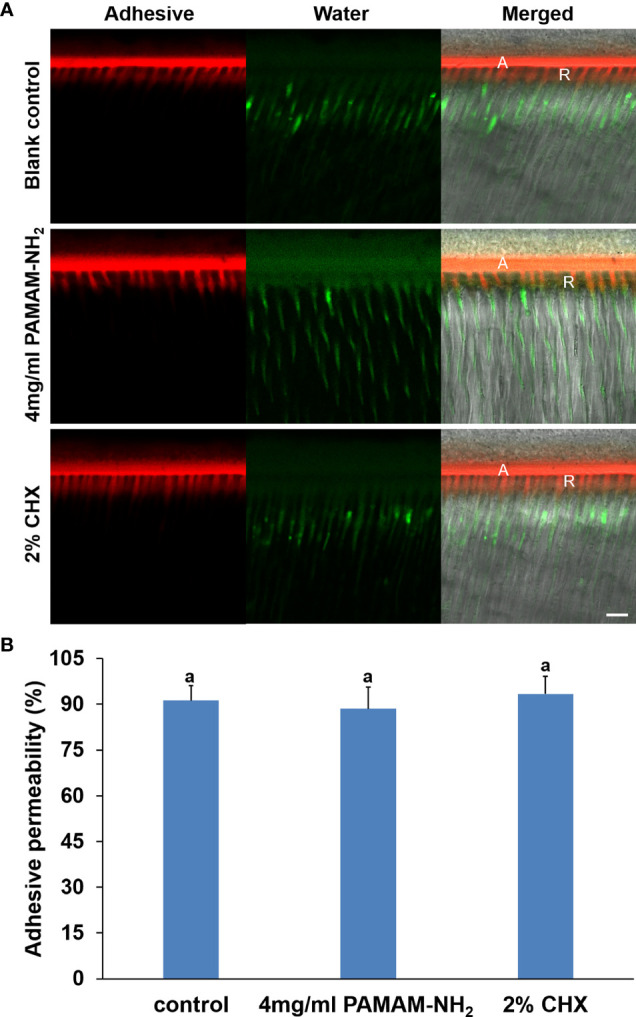
**(A)** Representative CLSM images illustrating adhesive permeability of resin–dentin interfaces with simulated pulpal pressure (20 cm water pressure) in the control, 4 mg/ml of PAMAM-NH_2_, and 2% CHX groups. Bars = 10 μm. A, adhesive layer; R, resin tag. Red channel, adhesive fluorescence; green channel, water containing fluorescent dye. **(B)** Bar chart comparing the relative adhesive permeability of the resin–dentin interfaces in the control, 4 mg/ml of PAMAM-NH_2_, and 2% CHX groups. Data are means ± SDs. Data obtained in the three groups (N = 6/group) were analyzed. Columns labeled with letters of the same case are not significantly different (p > 0.05). CLSM, confocal laser scanning microscopy.

A double-fluorescence technique was employed to evaluate the permeability of the resin–dentin interface created by the etch-and-rinse adhesive system under simulated pulpal pressure. The fluorescence representative images (separate channels; red for adhesive and green for water) of the permeability of adhesive are shown in **Figure 6A**. The adhesive infiltration data that are expressed as the relative percentage of red adhesive at the site of the dentinal tubules are presented in **Figure 6B**. In the control groups, the red adhesive sufficiently infiltrated into the dentinal tubules, as suggested by the presence of dense, branch-like resin tags at the resin–dentin interface. The relative percentages of the permeability were 91.2% ± 4.9%. At the bonded interface pretreated with PAMAM-NH_2_ or 2% CHX, the shape and depth of the resin tags were analogous to those groups in the control groups, reaching 88.5% ± 7.1% and 93.7% ± 5.8% permeability, respectively. There was no significant difference among these three groups in adhesive permeability values (p < 0.05).

**Figure 7:**
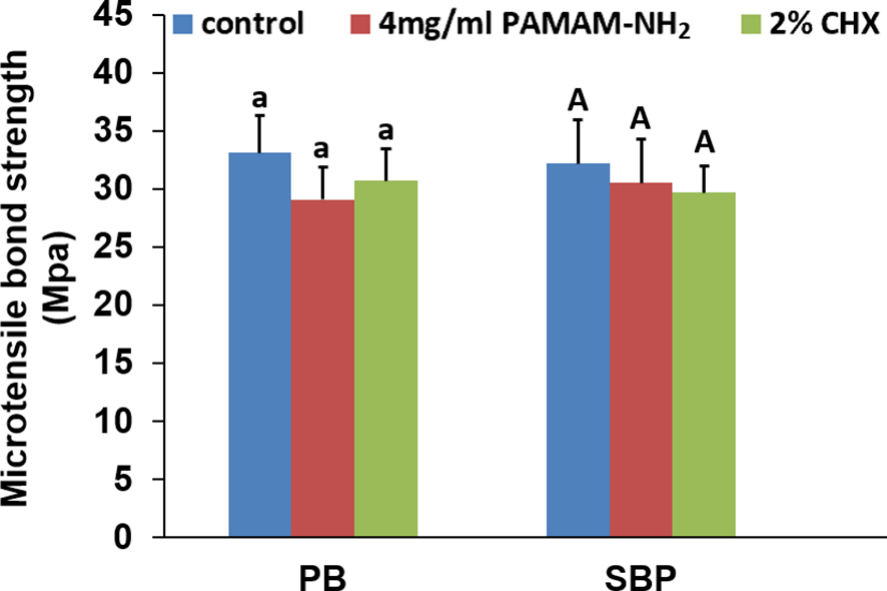
The microtensile bond strength of dentin created with the two different adhesives pretreated with different cavity cleansers. Data are means ± SDs. Data obtained in the three groups (N = 6/group) were analyzed. For PB, columns labeled with same lowercase letters are not significantly different (p > 0.05). For SBP, columns labeled with same uppercase letters are not significantly different (p > 0.05).

Tensile bond strength for each cavity cleanser group is shown in **Figure 7**. For both commercial adhesives PB and SBP, there was no significant difference among the control (without cavity cleanser), PAMAM-NH_2_, and 2% CHX groups (p > 0.05). Using PAMAM-NH_2_ cavity cleanser before adhesive application did not adversely affect the tensile bond strength of either adhesive. The two adhesive groups had similar failure mode distribution (**Table 2**). Low bond strength values tend to fail within the adhesive. Failure modes for all test beams exhibited obvious tendency of mixed failures, with a small distribution of cohesive failure in resin composite and cohesive failure in dentin.

**Table 2:** Percentage distribution of failure modes (A: adhesive failure; M: mixed failure; CC: cohesive failure in resin composite; CD: cohesive failure in dentin).

Failure mode	Prime & Bond NT^TM^			Adper^TM^ Single Bond Plus		
	Control	PAMAM-NH_2_	CHX	Control	PAMAM-NH_2_	CHX
A	10	11	8	11	14	7
M	42	39	40	41	39	45
CD	3	5	8	1	1	3
CC	5	5	4	7	6	5
Total	60	60	60	60	60	60

### 3.5 Cytotoxicity Assay

**Figure 8:**
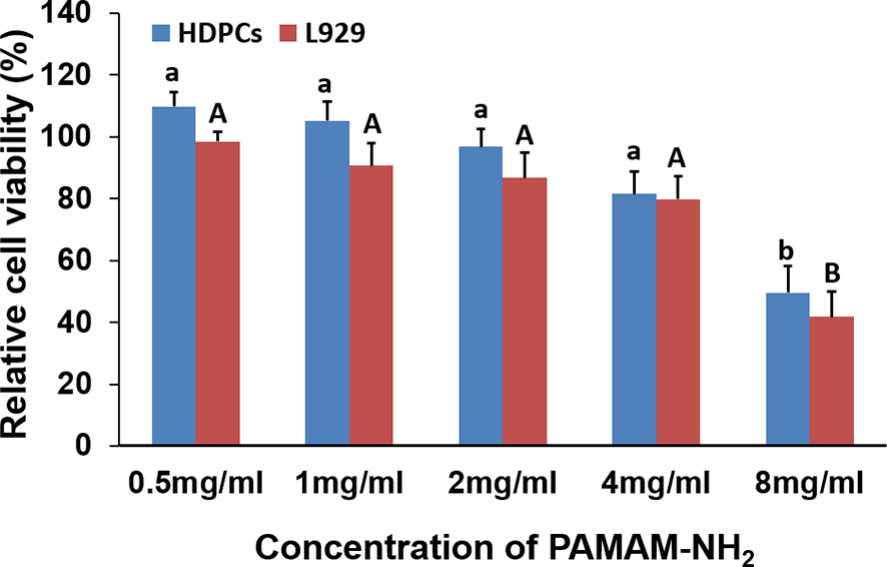
Cytotoxicity assay of PAMAM-NH_2_ to HDPCs and L929 at different concentrations by CCK-8 assay. Data are means ± SDs. Data obtained in the three groups (N = 6/group) were analyzed. For HDPCs, columns labeled with same lowercase letters are not significantly different (p > 0.05). For L929, columns labeled with same uppercase letters are not significantly different (p > 0.05). HDPCs, human dental pulp cells.

The cytotoxicity of PAMAM-NH_2_ on HDPCs and L929 at various concentrations from 0.5 to 8 mg/ml was evaluated using the CCK-8 assay (**Figure 8**). For both cells HDPCs and L929, there was no significant difference among the groups with PAMAM-NH_2_ at concentrations lower than 4 mg/ml (0.5 to 4 mg/ml) (p > 0.05). Good cell viability in the range of 80%–110% was observed with PAMAM-NH_2_ at concentrations lower than 4 mg/ml (0.5 to 4 mg/ml), which demonstrates that PAMAM-NH_2_ has low cytotoxicity at working concentrations.

## 4 Discussion

CHX is commonly recommended to be used as cavity cleanser in the clinic. Nevertheless, weak binding affinity for collagen and the leaching-out property make CHX short-lived, thus limiting its potential clinical applications. Therefore, it is in demand to develop a new cavity cleanser with long-term antimicrobial and anti-proteolytic activities.

PAMAM-NH_2_ dendrimer is categorized as one type of hyperbranched polymeric macromolecules and has been extensively investigated as a promising antibacterial agent (Castonguay et al., 2012; Mintzer et al., 2012). However, the dentinal tubules are filled with fluid. Intrapulpal pressure enables constant replenishment of intrinsic water from the pulp chamber to the dentin surface. Therefore, the binding capacity of PAMAM-NH_2_ to demineralized dentin is important to fulfill its long-term antimicrobial and anti-proteolytic effects. In this study, ATR-FTIR spectroscopy and CLSM of the demineralized dentin, after being conditioned with 4 mg/ml of PAMAM-NH_2_ or 2% CHX and after being washed with PBS, respectively, were first performed. From the present results, characteristic peaks of CHX or PAMAM-NH_2_ were clearly observed in ATR-FTIR spectra after dentin discs were conditioned with 2% CHX or PAMAM-NH_2_ (**Figure 1A**), indicating that both CHX and PAMAM-NH_2_ could bind to the demineralized dentin surface. However, the characteristic peaks of CHX disappeared in 2% CHX conditioning dentin surface, after being washed with PBS, demonstrating that 2% CHX had a weak binding capacity to demineralized dentin surface. In contrast, PAMAM-NH_2_-conditioned dentin surface retained large amounts of PAMAM-NH_2_ following washing, as suggested by the presence of the characteristic amide peaks. The results demonstrated that the binding capacity of PAMAM-NH_2_ on demineralized dentin surface was much stronger than that of 2% CHX, and the binding was strong enough to resist PBS washing. These results were also confirmed by CLSM. CLSM images (**Figure 1B**) showed that the yellow-green fluorescence was visible all over the surface of the FITC-labeled PAMAM-NH_2_ sample with an intensity value of 89.6% ± 3.5% (**Figure 1C**), while little fluorescence observed on the free FITC sample reaching 26.8% ± 5.7% fluorescence intensity after PBS washing. Thus, PAMAM-NH_2_ is considered to have a better binding capacity to demineralized dentin, as it can resist PBS washing. The results were consistent with a previous study, which also showed that PAMAM-NH_2_ had a good binding capacity to the demineralized dentin (Liang et al., 2015). The stronger binding capacity of PAMAM-NH_2_ over 2% CHX is likely attributed to its great number of functional groups. The external amine groups are positively charged groups, and the internal amide groups are negatively charged groups. These charged groups may help the molecule to bind to the collagen fibrils *via* electrostatic interactions (Liang et al., 2015). Thus, the first and third hypotheses that “the PAMAM-NH_2_ cavity cleanser has long-term inhibitory effects on bacteria and endogenous dentin proteases” are partially validated by the binding experiments.

Although PAMAM-NH_2_ has been extensively investigated as a promising antibacterial agent (Castonguay et al., 2012; Mintzer et al., 2012), there are just few reports whether PAMAM-NH_2_ has inhibitory effects on oral pathogens. *S. mutans* and *A. naeslundii* are cariogenic oral pathogens associated with secondary caries (Mo et al., 2010), which is described as a multifactorial infectious disease that is characterized by oral microbiome dysbiosis with the elevation of cariogenic bacteria (Tanner et al., 2016). *E. faecalis* is a common bacterium in filled root canals with persistent apical periodontitis (Wang et al., 2012). Because establishment of coronal seal with composite resin is frequently performed after placement of root fillings to prevent reinfection of the obturated canal space, the antibacterial activity of PAMAM-NH_2_ on *E. faecalis* was also evaluated (Guo et al., 2019). Therefore, these three microbes were chosen to evaluate the antibacterial properties of PAMAM-NH_2_ cavity cleanser. From the results of antibacterial activities, the antibacterial effect of PAMAM-NH_2_ was comparable with that of 2% CHX. Thus, the first hypothesis that “the PAMAM-NH_2_ cavity cleanser has long-term inhibitory effects on bacteria grown on dentin blocks” is totally validated. PAMAM-NH_2_ has a great number of positive charges on the protonated amino terminal groups on its exterior, which confers a strong affinity for bacterial surface with negative charges by electrostatic interactions. Such initial electrostatic interactions subsequently promote the disruption of anionic bacterial cell membranes and peptidoglycan, leading to leakage of cytoplasmic components and bacteria death (Castonguay et al., 2012; Mintzer et al., 2012; Gou et al., 2017). Due to its ability to damage bacteria through non-specific physical mechanisms rather than by targeting specific molecules (Wang et al., 2010), cationic PAMAM-NH_2_ dendrimer works against not only non-resistant bacteria but also currently antibiotic-resistant strains and is less likely to contribute to the development of bacteria resistance (Xue et al., 2013).

Dental plaque is a dynamic and complex ecosystem consisting of multispecies microbial communities. The development of dental caries is closely associated with imbalance in microbial equilibrium rather than a single pathogenic species (Filoche et al., 2010). Changes in the oral environment, such as food intake or saliva flow, may trigger a shift in dental plaque, in which acidogenic/aciduric species are selectively enriched at the expense of those less aciduric commensal residents (Filoche et al., 2010; Zheng et al., 2015). These changes lead to acid accumulation and subsequent pH declination, thus producing dental plaque with a more cariogenic composition. Several clinical studies confirmed that the diversity of the microbiota in carious lesions could be decreased by the establishment and dominance of acidogenic/aciduric species (Costalonga and Herzberg, 2014; Kianoush et al., 2014), and a higher proportion of *S. mutans* has been observed in lesion spots (Ge et al., 2008). In our study, with the stress from the PAMAM-NH_2_ cavity cleanser, the enriched acidogenic/aciduric species (e.g., *S. mutans* and *A. naeslundii*) were obviously inhibited and appeared to lose their dominant position, which has a potential effect in maintaining a healthy oral microbial equilibrium. Further biofilm composition studies and possible mechanism studies are required to support the potential biofilm species modulation of the presently developed bioactive PAMAM-NH_2_ cavity cleaner.

The hybrid layer remains the weakest link within the bonded interface due to its degradation *via* endogenous dentin proteases. They become exposed and activated during the acid-etching and adhesive placement steps of contemporary bonding procedures, which contributes to the degradation of exposed collagen fibrils within the hybrid layers. Therefore, our present study also aimed to explore whether PAMAM-NH_2_ can inhibit endogenous MMP in the dentin matrix. Soluble rhMMP-9 was employed for examining the potential inhibitory effect of PAMAM-NH_2_ by Sensolyte assay kits. The results of the quantitative assay demonstrated that the extent of rhMMP-9 inhibition was proportional to PAMAM-NH_2_ concentrations. The anti-MMP-9 activities of PAMAM-NH_2_ at concentrations higher than 4 mg/ml (4 to 16 mg/ml) were comparable with those of the GM6001 control group (p > 0.05). Hence, the second hypothesis that “the PAMAM-NH_2_ cavity cleanser has inhibitory effects on soluble MMP-9 activities” is validated. However, this experiment confirmed that PAMAM-NH_2_ has inhibitory effects on exogenous rhMMP-9. Its effect on endogenous MMP-9 embedded within collagen matrix should also be investigated. In the present study, *in situ* zymography was employed to detect the proteolytic activity of the endogenous MMP-9 directly within dentin hybrid layers (Frederiks and Mook, 2004; Gou et al., 2018b). According to the concentrations of PAMAM-NH_2_ tested from antibacterial activities and inhibitory effects on exogenous rhMMP-9, the concentration of 4 mg/ml of PAMAM-NH_2_ was used for the following experiments. For the control groups, extensive green fluorescence was detected within the hybrid layers, indicating strong gelatinolytic activity. In contrast, dentin slabs pretreated with 4 mg/ml of PAMAM-NH_2_ exhibited weak gelatinolytic activity within the hybrid layers after incubation for 48 h, which is significantly lower than that of the control group. Thus, the third hypothesis that “the PAMAM-NH_2_ cavity cleanser has long-term inhibitory effects on endogenous dentin proteases” is totally validated.

Although the functional mechanism of inhibitory effects of PAMAM-NH_2_ on dentin proteases is still not clear, several factors may have contributed to the inhibitory effect. The catalytic domains of MMPs contain cysteine-rich sites, including negatively charged glutamic acid residues (Tezvergil-Mutluay et al., 2011). PAMAM-NH_2_ may bind electrostatically to the negatively charged glutamic acid residues with a great number of positive charges on the protonated amino groups on its exterior. This non-specific binding can change the configuration of the catalytic site of the MMPs by electrostatic interaction with the negatively charged glutamic acid residues, sterically blocking the active site and inhibiting the activation of MMPs. Additionally, amine-terminated dendritic polymers were reported as a multifunctional chelating agent for heavy metal ion removals (Mohseni et al., 2019). Accordingly, we surmise that the inhibitory effect of PAMAM-NH_2_ on MMPs is potentially related to its potency of chelation on Zn^2+^ and Ca^2+^. MMPs are a family of Zn- and Ca-dependent enzymes (Zitka et al., 2010). PAMAM-NH_2_ may chelate Zn^2+^ or Ca^2+^ that can be bound to the Zn^2+^- and Ca^2+^-active sites of the catalytic domain of MMPs (Wu et al., 2019), which is also conductive to inhibiting MMP activities.

Adhesive infiltration into the dentinal tubules is paramount for preserving the integrity of resin–dentin bonding. To evaluate the effect of PAMAM-NH_2_ on the adhesive permeation and morphology of the resin–dentin interface, the bonded dentin interface was observed by a double-fluorescence CLSM technique. From the present results, the resin tag shared a morphological similarity in both experimental and control groups. The quantitative analysis of permeability also demonstrated that PAMAM-NH_2_ as a cavity cleanser did not decrease the infiltration of adhesive monomers.

The dentin tensile bond strengths were also performed to evaluate whether the application of PAMAM-NH_2_ adversely affects the tensile bond strength of commercial adhesive. The results showed that pretreatment of dentin surface with PAMAM-NH_2_ or 2% CHX had no adverse effect on the dentin bond strength. Thus, the fourth hypothesis that “treatment of dentin surface with PAMAM-NH_2_ cavity cleanser does not adversely affect dentin bond strength” is validated.

In our study, the relative cell viability in the range of 80%–110% was observed with PAMAM-NH_2_ at concentrations equal to or lower than 4 mg/ml (0.5 to 4 mg/ml), which is considered non-cytotoxic (Zou et al., 2011; International Organization for Standardization, 2009) and has potential clinical application at working concentration.

## 5 Conclusion

Within the limitations of the present study, it may be concluded that PAMAM-NH_2_ cavity cleanser developed in this study could provide simultaneous long-term antimicrobial and anti-proteolytic activities for eliminating secondary caries that results from a dysbiosis in the oral microbiome and preventing hybrid layers from degradation due to its good binding capacity to dentin collagen matrix, which are crucial for the maintenance of resin–dentin bond durability. Although the price of the commercially available cavity cleaner–2% CHX may be slightly lower than that of PAMAM-NH_2_, long-term antibacterial and anti-proteolytic activities may give PAMAM-NH_2_ an advantage over CHX.

## Data Availability Statement

The original contributions presented in the study are included in the article/supplementary material. Further inquiries can be directed to the corresponding authors.

## Ethics Statement

The studies involving human participants were reviewed and approved by Clinical Scientific Research Ethics Committee of Hospital of Stomatology, Lanzhou University. The patients/participants provided their written informed consent to participate in this study.

## Author Contributions

YG contributed to the conception, design, data acquisition, analysis, and interpretation and drafted and revised the manuscript. WJ contributed to the data acquisition and interpretation and drafted the manuscript. YaH contributed to the data acquisition and analysis and drafted the manuscript. YL, RS, YuH, ZW, and JL contributed to the design, data analysis, and interpretation and drafted the manuscript. BL contributed to the data analysis and interpretation and critically revised the manuscript. All authors contributed to the article and approved the submitted version.

## Funding

This work was supported by the National Natural Science Foundation of China grant 82001034, Natural Science Foundation in Gansu Province of China grant 20JR10RA595, Fundamental Research Funds for the Central Universities of Lanzhou University grant lzujbky-2020-53, and School/Hospital of Stomatology, Lanzhou University grant lzukqky-2019-y15 (YG).

## Conflict of Interest

The authors declare that the research was conducted in the absence of any commercial or financial relationships that could be construed as a potential conflict of interest.

## Publisher’s Note

All claims expressed in this article are solely those of the authors and do not necessarily represent those of their affiliated organizations, or those of the publisher, the editors and the reviewers. Any product that may be evaluated in this article, or claim that may be made by its manufacturer, is not guaranteed or endorsed by the publisher.
